# Projection of corn production and stover-harvesting impacts on soil organic carbon dynamics in the U.S. Temperate Prairies

**DOI:** 10.1038/srep10830

**Published:** 2015-06-01

**Authors:** Yiping Wu, Shuguang Liu, Claudia J. Young, Devendra Dahal, Terry L. Sohl, Brian Davis

**Affiliations:** 1 ASRC Federal, contractor to U.S. Geological Survey (USGS) Earth Resources Observation and Science (EROS) Center, Sioux Falls, SD 57198, USA; 2 U.S. Geological Survey (USGS) Earth Resources Observation and Science (EROS) Center, Sioux Falls, SD 57198, USA; 3 Innovate! Inc., contractor to the USGS EROS Center, Sioux Falls, SD 57198; 4 Stinger Ghaffarian Technologies, contractor to the USGS EROS Center, Sioux Falls, SD 57198

## Abstract

Terrestrial carbon sequestration potential is widely considered as a realistic option for mitigating greenhouse gas emissions. However, this potential may be threatened by global changes including climate, land use, and management changes such as increased corn stover harvesting for rising production of cellulosic biofuel. Therefore, it is critical to investigate the dynamics of soil organic carbon (SOC) at regional or global scale. This study simulated the corn production and spatiotemporal changes of SOC in the U.S. Temperate Prairies, which covers over one-third of the U.S. corn acreage, using a biogeochemical model with multiple climate and land-use change projections. The corn production (either grain yield or stover biomass) could reach 88.7–104.7 TgC as of 2050, 70–101% increase when compared to the base year of 2010. A removal of 50% stover at the regional scale could be a reasonable cap in view of maintaining SOC content and soil fertility especially in the beginning years. The projected SOC dynamics indicated that the average carbon sequestration potential across the entire region may vary from 12.7 to 19.6 g C/m^2^/yr (i.e., 6.6–10.2 g TgC/yr). This study not only helps understand SOC dynamics but also provides decision support for sustainable biofuel development.

The carbon sequestration potential of terrestrial ecosystems (i.e., net carbon flux from the atmosphere to the land) has attracted much attention especially in today’s world, which is characterized by rising greenhouse gas emissions and the resulting global warming^1^,^2^,^3^. Terrestrial ecosystems can convert atmospheric carbon dioxide (CO_2_) into SOC through plant photosynthesis and returning the crop residue into soil, but soil respiration may offset this atmosphere-to-land carbon exchange in some degree^4^,^5^,^6^. Global soils were estimated to contain 1550 Gigatons (Gt) of SOC, which is about double the carbon in the atmosphere (760 Gt)^7^,^8^ and also greater than the amount in living vegetation (560 Gt)^8^,^9^. The SOC density (carbon amount per unit area) can affect soil fertility and thus influence plant production, agronomic productivity, and the carbon sink rate^8^,^10^,^11^,^12^. Therefore, the SOC pool is a critical component for storing carbon and plays a key role in the global carbon cycle, requiring an intensive understanding of SOC dynamics.

Global environmental changes, including an elevated atmospheric CO_2_ concentration, climate change and land-use change, have a profound impact on terrestrial ecosystems and SOC dynamics^1^,^2^,^4^,^13^. Although enrichment of CO_2_ concentration may enhance plant production^14^,^15^, this direct physiological effect may decrease with increasing CO_2_ and diminish due to changes in climatic factors^4^,^5^,^16^. The enhancement effects of climate change on global vegetation productivity were reported^17^,^18^; however, there was also a concern that the changing climate may reduce the carbon uptake because of the decrease in vegetation productivity for some areas, prolonged dry days and fire seasons due to changes in precipitation distribution^19^,^20^, and an increase in soil respiration due to global warming^5^,^21^. Therefore, the potential of the terrestrial carbon sink would not persist indefinitely at high levels^1^,^4^. For example, the U.S. carbon sink was projected to slow down over the 21st century, from 0.33 PgC/yr in the 1980s to 0.21 PgC/yr by 2050 and to 0.13 PgC/yr by 2100^1^.

On the other hand, land-use/management changes (e.g., type change, crop rotation, and residue management) can strongly affect SOC dynamics and thus may result in changes to the carbon sequestration potential. The conversion of natural vegetation to cultivated use is the primary cause of SOC loss, which can be attributed to the reduced inputs of organic matter, increased decomposability of residue, and decreased physical protection from wind/water erosion, and soil disturbance by tillage^7^,^8^,^9^. The Energy Independence and Security Act (EISA) of 2007, which aims to increase the energy efficiency and availability of renewable energy in the United States and reduce U.S. dependence on foreign oil, requires fuel producers to use at least 36 billion gallons from biofuels by 2022^22^. In 2011, more than 40% of corn grain in the United States was channeled to ethanol processing^23^,^24^. By 2030, cellulosic feedstock was estimated to be one billion megagrams (Mg) to meet the target of bioenergy production and most will be from crop residues^25^,^26^. Therefore, rapid biofuel development brings an expectation of expanding corn cultivation for producing corn kernel-based ethanol and corn stover harvesting for producing cellulosic biofuel^27^,^28^. These two potential measures (i.e., increase in corn planting area and corn stover removal) may cause adverse environmental consequences, including depletion of SOC with aggravating emissions^29^,^30^. Under these circumstances, the Temperate Prairies of the U.S. Great Plains may be strongly affected by increased biofuel production because this region has the most fertile soils and the highest percentage of cropland, compared to other areas of the Great Plains^31^.

The objective of this study was to project corn production and SOC dynamics across the Temperate Prairies ecoregion as influenced by climate and land-use changes over the coming decades using a well-established biogeochemical model—Erosion and Deposition Carbon Model (EDCM)^32^. Because corn stover is considered to be one of the candidates for second generation (cellulosic) biofuel feedstock, we also investigated the impacts of different corn stover removal rates on SOC dynamics over the corn planting area and the entire region, and identified a reasonable upper limit of stover harvesting to ensure sustainability.

## Results

### Model evaluation

**Figure 1:**
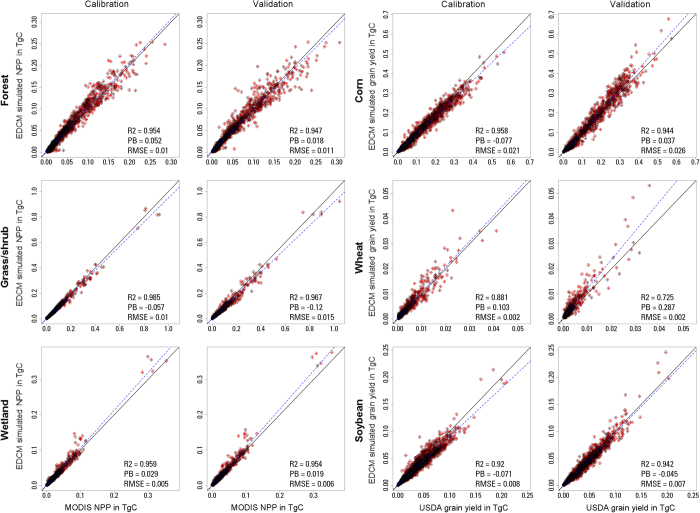
Scatter plots of county-based annual total NPP of MODIS and EDCM for forest, grass/shrub, and wetland (left two columns) and grain yield of USDA and EDCM for corn, wheat, and soybean (right two columns) during the regional calibration (2001–2005) and validation (2006–2010 for NPP and 2006–2009 for grain yields) periods. One data point represents the annual total NPP or grain yield for a county, and unit TgC is 10^12^ gC.

The 2001–2009 observed grain yield and the 2001–2010 MODIS NPP data were used to evaluate the model performance for the calibration and validation periods (see Methods). Figure 1 shows the county-based scatter plots, which clearly demonstrated the good model performance in NPP simulation for non-crop land covers (forest, grass/shrub, and wetland) and grain yield simulation for crop land covers (major crop species including corn, wheat, and soybean). For example, the R^2^ (Coefficient of Determination) varied from 0.947 to 0.985 for non-crop land covers during both calibration and validation periods, with absolute Percent Bias (|PB|) less than 12%. For the three major crop species, R^2^ ranged between 0.725 and 0.958, and |PB| was no larger than 29% during the calibration and validation periods. For corn, which is the focus of the study, R^2^ reached 0.958 and 0.944 for calibration and validation, respectively, with |PB| less than 8%. Therefore, both graphical comparison and statistical measures indicated that model simulations were in good agreement with observations for a variety of ecosystems.

### Corn production

**Figure 2:**
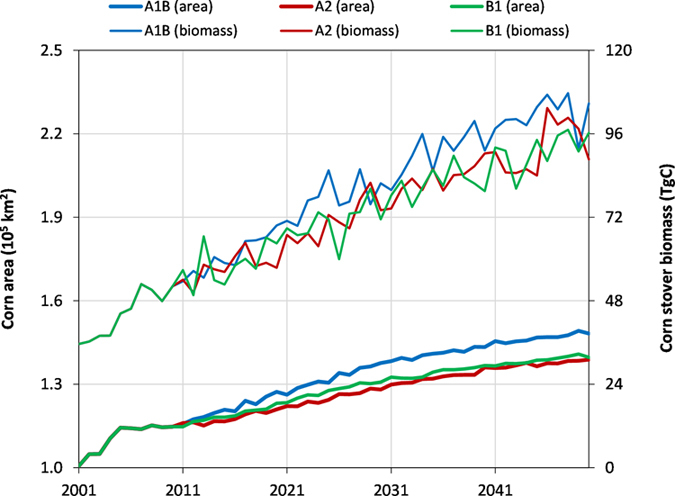
Annual corn area and simulated corn stover biomass over the Temperate Prairies under three scenarios (A1B, A2, and B1) during the 50-year (2001–2050) simulation period. TgC is 10^12^ gC.

Figure S.1 in the Supporting Information showed the annual proportions of the land-use classes in the Temperate Prairies from 2001 to 2050. The cropland was projected to increase from 58.6% in 2001 to 59.8% and 59.5% under the A1B and A2 scenarios, respectively, by 2050. However, cropland decreased to 57.4% for B1 (Figure S.1). The changes referred to an overall trend, and further details about the land-use changes between scenarios can be found in previous publications^33^,^34^. Because corn production and stover harvesting are the focus of the current study, the annual time series of corn area based on the land-use projection by FORE-SCE and species allocation (see Methods) are summarized in Fig. 2. The corn area was projected to rise under the three scenarios, with A1B showing the greatest increase rate and A2 the least. The increase rate became a little lower in the last 20 years (2031–2050) compared to the first 20 years (2011–2030) under any scenario (Fig. 2). For A1B, the corn area was projected to expand from 1.15 × 10^5^ km^2^ (about 22% of the entire region) in 2010 to 1.48 × 10^5^ km^2^ (about 28% of the entire region) by 2050, with a relative increase of 28% during the 40 years; whereas this expansion rate was a little lower (about 21% of relative increase) for A2 and B1 (Table 1 and Fig. 2). Compared to the increase of cropland area (absolute increase of 1.2% of entire region), the absolute increase of corn area (6% of the entire region) could be mainly attributed to an increased frequency of corn production in the crop rotation system (i.e., a decreased crop rotation for other grains like soybean).

With the increase in corn planting area, the corn production (both grain and stover) was also expected to rise. As shown in Fig. 2, the overall annual biomass of corn stover was increasing for the coming decades. However, this increase in production is not proportional to (larger than) the increase of corn area due to the enriched CO_2_ effects and projected biological enhancement of crop production (see Methods). Figure 2 also indicates that the annual total production varied from year to year, which can be attributed to the changes in climate and planting area and locations (land-use change and crop rotation). By 2050, for example, the annual stover biomass could be as high as 104.7 TgC under the A1B scenario, whose increase of 101% relative to 2010 (52.2 TgC) was much higher than that for corn planting area (about 28% as given earlier) (Table 1 and Fig. 2). For B1 and A2, the stover biomass was estimated to be 96.3 and 88.7 TgC, (i.e., 85% and 70% increase based on 2010), respectively (Table 1 and Fig. 2). Compared to the current 0.45 kg C/m^2^ in 2010, the annual corn stover biomass production could range between 0.64 and 0.71 kg C/m^2^ (or 6.4–7.1 Mg C/ha, depending on scenarios) in this area by 2050 (Table 1).

### Soil organic carbon

**Figure 3:**
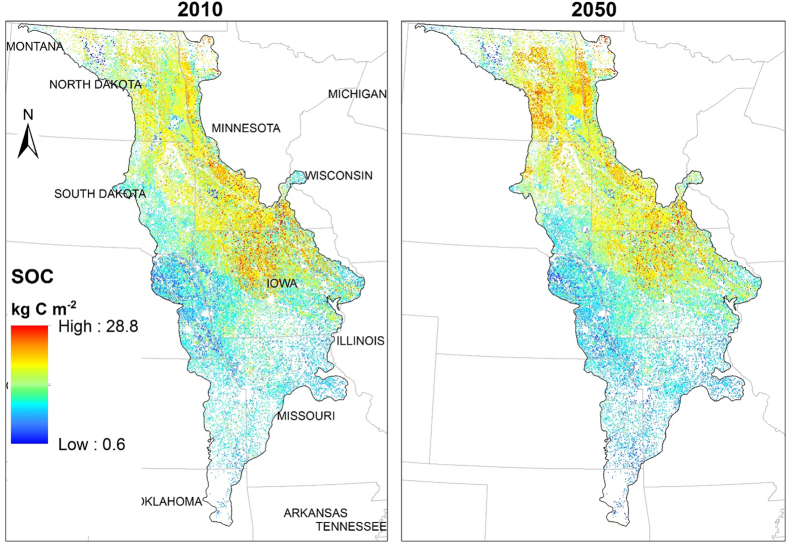
The simulated spatial soil organic carbon (SOC) for corn areas in the Temperate Prairies under the A1B_50 scenario (i.e., A1B climate and land use with 50% of corn stover harvesting rate) for year 2010 and 2050. The ‘corn areas’ refer to a mask containing pixels where corn cultivation occurred in any year between 2010 and 2050. The maps were created using ArcGIS10.2.

The EDCM model was able to derive the annual SOC storage for the entire region (with 10 × 10 sampling approach as stated in the Methods). Because corn cultivation is the focus of the study, it would be specific to isolate the spatial SOC storage map and changes on the corn fields only. However, corn areas could change every year due to the land-use change (e.g., from grass to corn to grass) and crop rotations (e.g., corn-soybean-wheat-corn). To solve this issue, we extracted all the corn pixels where corn was planted from 2010 through 2050 to generate a fixed ‘corn-area’. Using this ‘corn-area’ as a mask, as shown in Fig. 3, we presented the spatial SOC storage for 2010 (base year) and 2050 using the A1B_50 scenario (A1B climate and land use with 50% corn stover harvesting, see Table 2) as an example. The visual comparison between the two years demonstrated the accumulation of SOC particularly in the northern part of the region between 2010 and 2050 (i.e., from central Iowa to the northern border, involving the northern part of Iowa, South Dakota, North Dakota, and Minnesota). The comparison also indicated that the overall average SOC density (i.e., amount of SOC per unit area) across the corn area could still increase by 8% during the 40 years under the A1B scenario with 50% stover harvesting, from 6.31 kg C/m^2^ in 2010 to 6.81 kg C/m^2^ in 2050 (Fig. 3 and Table 1).

**Figure 4:**
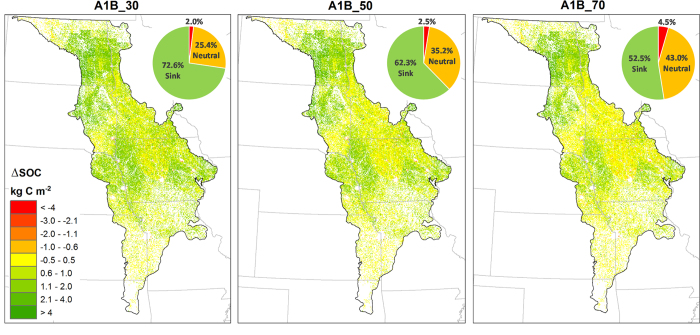
The simulated spatial change of soil organic carbon (SOC) between 2010 and 2050 for corn areas in the Temperate Prairies under three scenarios (A1B_30, A1B_50, and A1B_70). Definitions of these scenarios are given in Table 2. The ‘corn areas’ refer to a mask containing pixels where corn cultivation occurred in any year between 2010 and 2050. The embedded pie chart showed the percentage of area identified as carbon sink (green), neutral (orange), and source (red) under each scenario. The maps were created using ArcGIS10.2.

To illustrate the impacts of corn stover harvesting on SOC dynamics, we compared the spatial SOC maps between 2010 and 2050 for the proposed three stover harvesting schemes (30%, 50%, and 70% stover removal rates) under the A1B scenario (i.e., A1B_30, A1B_50, and A1B_70 in Table 2). Figure 4 illustrates the spatial change of SOC between 2010 and 2050 (i.e., SOC in 2050 minus SOC in 2010). Based on the temporal change of SOC for a given period (e.g., 40 years), we classified a specific site or area as a carbon sink (positive), source (negative), or neutral (zero). Considering the uncertainties that exist in modeling, we defined the small change ranging between –0.5 and 0.5 kg C/m^2^ (instead of exact zero) during the 40-year projection period (2011–2050) as neutral (i.e., –12.5 to 12.5 g C/m^2^/yr). For A1B and 30% of stover harvesting (i.e., A1B_30), as shown in Fig. 4, the carbon sink and source areas accounted for about 72.6% and 2%, with the remaining 25.4% of the corn area as carbon neutral. The carbon sink area decreased to 62.3% and 52.5% for A1B_50 and A1B_70, respectively. Comparing A1B_70 with A1B_30, the area identified as a carbon source more than doubled, and the area evaluated as neutral increased by 69% when raising the stover removal rate from 30% to 70% (Fig. 4). In summary, the area with SOC sequestration potential would become less with more corn stover being harvested, and the identified vulnerable areas were also illustrated (Fig. 4).

**Figure 5:**
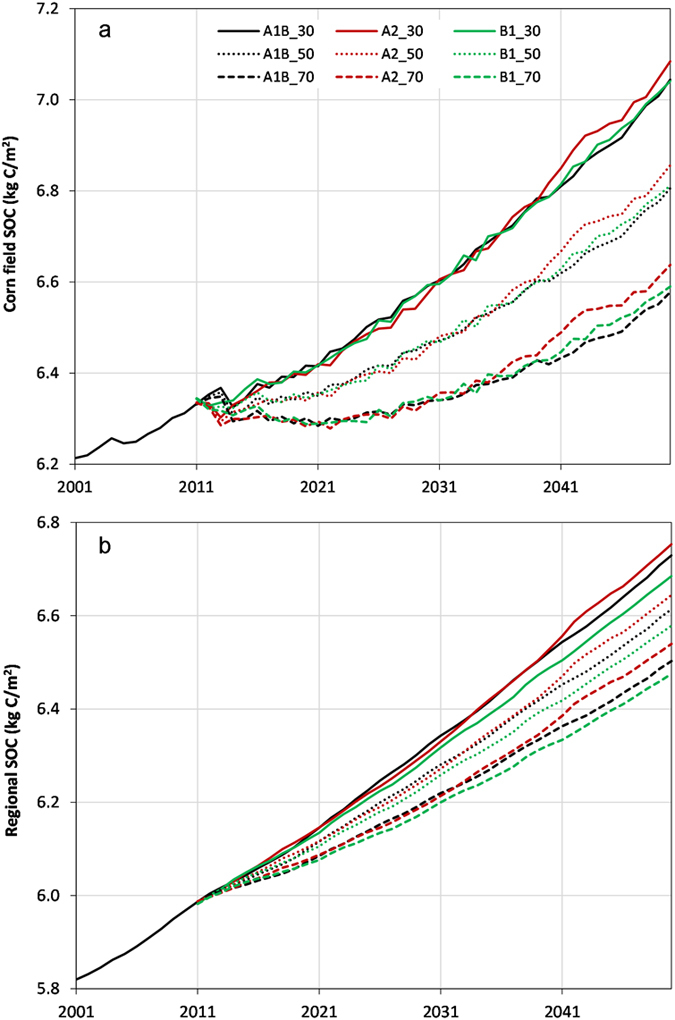
Annual time series of average soil organic carbon (SOC) for the corn field areas (**a**) and the entire region (**b**) under the nine scenarios during the 50-year (2001–2050) simulation period.

To evaluate the overall SOC sequestration capacity, Fig. 5 shows the annual time series of average SOC density under all nine scenarios (5a for corn area only and 5b for the entire region). From Fig. 5a, the average SOC for the corn planting area varied slightly between scenarios for a given corn stover harvesting rate. Using the scenario A1B_30 as an example, the average SOC for corn planting area could increase from 6.31 kg C/m^2^ in 2010 to 7.04 kg C/m^2^ in 2050, which was equivalent to sequestering 18.3 g C/m^2^ per year. However, this sequestration potential could decline to 12.4 g C/m^2^/yr and 6.7 g C/m^2^/yr for 50% and 70% corn stover harvesting rates (i.e., A1B_50 and A1B_70), respectively. Taking into account the corn area in 2050 (1.48 × 10^5^ km^2^) as projected under the scenario A1B, the total amount of SOC sequestration potential across the corn area could be 2.7 TgC/yr, 1.8 TgC/yr, and 1.0 TgC/yr, respectively, for the three corn stover harvesting schemes. Table 1 indicates that the average annual carbon sink potential across the corn could range between 6.7 and 19.3 g C/m^2^/yr (i.e., 1.0–2.9 TgC/yr) depending on scenarios.

For the first 10 years (2011–2020) of the projection period, although 30% corn stover harvesting scenarios may still support the corn area as a gentle carbon sink (e.g., an average of 8 g C/m^2^/yr for A1B), the 50% scenarios may become neutral and the 70% scenarios would lead to a carbon source (e.g., an average of –3.0 g–C/m^2^/yr for A1B) (Fig. 5a). Rates of corn stover removal over 50% would result in an overall SOC loss across the corn planting area in the near future. However, this SOC loss would not be a concern over time due mainly to the enriched CO_2_ effects and biological enhancement of corn production.

In addition, we examined the evolution of SOC across the entire region under all nine scenarios. As shown in Fig. 5b, the annual time series of SOC demonstrated that this region would still be a carbon sink. However, the projected magnitude of SOC density by 2050 varied between scenarios, with the lowest (6.48 kg C/m^2^) for B1_70 and the highest (6.75 kg C/m^2^) for A2_30, indicating an average annual carbon sink of 12.7–19.6 g C/m^2^/yr during the 40 years (2010–2050). This sequestration capacity (i.e., 6.6–10.2 TgC/yr in total amount) of the entire region is close to the U.S. CO_2_ emissions from natural gas systems in 2011 (32 Tg CO2 eq.)^35^. In terms of the SOC density, there is a potential range of 0.28 kg C/m^2^ (maximum minus minimum) in predicting the 2050 amount. Considering the huge area of the Temperate Prairies, the 2050 SOC storage may be between 3376.6 TgC and 3521.4 TgC, indicating a potential variation range of 145 TgC among all nine scenarios.

## Discussion

This study shows that the potential production of corn stover biomass may increase substantially from 2010 through 2050 (i.e., 71–101% depending on scenarios). Scenario A1B demonstrated the highest corn cultivation, which is reasonable because this scenario is marked by high technological change and strong energy demands, including projected increases in the use of traditional biofuels (e.g., corn ethanol) and widespread adoption of the use of cellulosic feedstock (e.g., corn stover) for biofuels. In addition, the scenario A1B assumes moderate population growth, very high economic growth, and a standardization of global living standards; all these factors are likely to increase pressures on agricultural land use in the Midwestern United States^36^. The scenario B1 has the same global population assumptions as A1B. However, lower economic growth and more of a focus on environmental conservation resulted in lower overall expansion of corn area. The scenario A2 assumed extremely high population growth, but lower technological innovation and economic growth than A1B.

In terms of the unit-area corn production (either grain yield or stover biomass), the relative increase was about 42–58% over the 40 years (2010–2050)—from 0.45 kg C/m^2^ in 2010 to 0.64–0.71 kg C/m^2^ (depending on scenarios) in 2050. The average annual increase was 1.1–1.4%, which was within the baseline (1% annual increase) and high-yield (2% annual increase) assumptions by Billion Ton^26^. Using 2030 as an example, our estimated 5.5–5.9 Mg C/ha for corn production was also reasonable when compared to the projected 2030 yield—about 4–6 Mg dry biomass/acre (i.e., 5–7 Mg C/ha) in this region^26^.

We observed that the identified potential carbon source areas are characterized by the high SOC levels (e.g., especially in central to northern Iowa) (Fig. 3 and Fig. 4). This finding agreed with previous studies that showed soil with high SOC levels may tend to release carbon^10^,^37^ because SOC tends to progress toward its equilibrium level which depends on the land use and management practices applied for a given site with a given climate condition^38^,^39^. Land-use change could alter the equilibrium level, and thus extensive stover harvesting in high SOC areas (vulnerable areas) would likely result in substantial release of carbon. Moreover, our studies indicated that over 50% stover removal could cause the SOC loss across the corn planting area in the near future, and this critical stover harvesting rate agreed with other studies^40^,^41^. Additionally, our simulated regional average carbon sequestration potential of 19.6 g C/m^2^/yr under scenario A2_30 also makes sense when compared to the reported 26.6 g C/m^2^/yr for the entire Great Plains without stover removal^42^.

Although the projected 2050 SOC density seemed to have a small range (0.28 kg C/m^2^) among all the nine scenarios, the SOC storage in 2050 may have a substantial range (145 TgC). This amount is equivalent to 10% of the U.S. CO_2_ emissions from fossil fuel combustion in 2011 (5,277 Tg CO_2_ eq.)^35^, or close to the estimated net carbon sink capacity (135–205 TgC) in Europe’s terrestrial biosphere^43^. The substantial variation can be attributed to different climate and land-use projections combined with different corn stover harvesting schemes. Therefore, environmental and management changes, especially crop residue management (see Fig. 5), may produce a prominent difference in SOC dynamics, suggesting the need for better management practices in enhancing the carbon sequestration potential of farmlands.

Our projected corn production demonstrated that the expansion of corn cultivating area, CO_2_ enrichment, and potential biological enhancement could make the corn stover biomass reach 0.64–0.71 kg C/m^2^ (depending on scenarios) by 2050, suggesting as high as 104.7 TgC stover in total in the Temperate Prairies. This finding may indicate a potential substantial increase in supply of both corn kernels for traditional corn ethanol production and corn stover for the advanced biofuel production by the mid-century. Further, we found that 50% could be a reasonably high corn stover harvesting rate because a rate beyond this critical value could lead to an overall loss of SOC sequestration potential from the corn planting areas within about a decade. However, these areas would again become a sink owing to the projected corn-production potential. Although a lower stover removal rate is better for maintaining or enhancing SOC, the reduction of stover biomass supply per unit area may compromise the efficiency of biofuel production (e.g., potential increase in transportation cost for collecting the same amount of stover biomass). Therefore, a balance between mitigating greenhouse gas emissions and pursuing a high efficiency of biofuel production is needed for decision making. Further evaluation into the socio-economic aspects of corn stover as feedstock for cellulosic ethanol is required for deriving an appropriate and optimal rate. Nonetheless, 50% may be a reasonable upper limit in order for maintaining SOC level and fertility and carbon sequestration potential, at least at the current stage and the coming 10 years. Although the potential cap of stover biomass produced from this region was estimated to be as high as 52 TgC by 2050 (under scenario A1B), the practical harvesting amount for biofuel production could be lower because not all corn fields are within the collection radius of refinery plants. Also, 50% stover harvesting was based on the overall SOC dynamics across the corn area, but this rate or even a lower rate does not guarantee a net carbon sink everywhere. For example, as shown in Fig. 4, there are some locations that may release carbon even with a much lower stover-harvesting rate of 30%. In this case, it would be beneficial to derive spatially varying stover removal rates, especially for soils with a high SOC content, which may alleviate the carbon emission to some degree. Additionally, best management practices (e.g., intensified manure application) deserve to be evaluated to determine their effects in compensating the reduction of SOC and fertility caused by corn stover harvesting. Overall, this study is valuable for understanding the potential evolution of SOC in this highly agricultural ecoregion and can be a useful guide for decision-makers to seek sustainable biofuel production and farming practices.

Although this study with the application of a well-established biogeochemical model is recognized in terms of study design and data analysis, there were a few limitations that need to be stated. Although EDCM can simulate impacts of climate change, it may not simulate well the damaging effects of extreme weather such as high temperatures and droughts in a short time scale (e.g., day or week) because it is a monthly-scale model. Soil erosion and deposition may lead to carbon loss and carbon gain (burial)^32^, depending on the rate of SOC replacement at the eroding sites, changes in the reactivity of SOC due to transport/burial (i.e., the fate of eroded SOC), and the rates of erosion and deposition. There is still a controversy about the net effect of erosion (source or sink) at large (global/regional) scale^12^,^44^,^45^,^46^,^47^,^48^. In this study, we did not activate the soil erosion/deposition function due to the lack of extensive spatial data EDCM required, and this may lead to a slight bias of SOC simulations. From this dispute, however, it is clear that quantifying the spatially explicit effects of soil erosion/deposition and the resulting carbon movement on SOC dynamics is a good topic and deserves intensive investigations in our further work. Application of numerical models usually involves parameter optimization, and thus the analysis of parameter uncertainty and quantification of its effects on model output (e.g., SOC) are attractive and challenging in the modeling field^49^ especially for large-scale studies^50^,^51^. From our experience with parameter uncertainty analysis^49^,^52^,^53^, the R packages such as Flexible Modeling Environment (FME) could be a potential tool for this purpose. In addition to the parameter uncertainty, there are other uncertainties to consider such as those existing in the initial SOC data (from SSURGO and STATSGO), the scenario climate data (from GCMs), crop management data, and the implicit assumption of similar occurrence of pests and diseases in the future. Clearly, it is valuable to investigate the above important issues in future work.

## Methods

### Study area

The Temperate Prairies ecoregion (Figure S.2 in the Supporting Information) (Ecoregion Level II)^54^, is located in the north-central United States, and together with the other two adjacent regions—the West-Central Semi-Arid Prairies and the South-Central Semi-Arid Prairies, compose the Great Plains. The Temperate Prairies, with an area of 521,455 km^2^, was once covered with natural grasslands (mainly tall-grass prairie) that supported abundant and highly specialized plant and animal communities^55^. The climate is moderately humid, but long periods of cold temperatures prevail with high winds. The annual average precipitation is about 780 mm, and the annual average air temperature is about 9 °C. The prairie soils are commonly deep, and most of the region was originally highly fertile—one of the most productive soils in the world. Thus, many native prairie vegetation types have been radically transformed, and 60–70% of the region (see Figure S.2) has been plowed and cultivated, mainly for corn/soybean cultivation, which is higher than the other two ecoregions of the Great Plains^42^,^55^. Part of this region also belongs to the U.S. Corn Belt agro-ecosystem^54^, and the entire Temperate Prairies covers more than one-third of the U.S. corn acreage. Land-use change and intensive management practices have caused significant impacts on water, carbon and nutrient cycles of farmlands^56^.

### GEMS-EDCM modeling framework

The General Ensemble Biogeochemical Modeling System (GEMS) framework, developed by the U. S. Geological Survey (USGS), provides spatially explicit biogeochemical simulations over large areas by integrating well-established ecosystem biogeochemical models and numerous spatial databases^57^. GEMS is a regional-level biogeochemical simulation system assimilating spatially dynamic databases such as climate, land use, management, and disturbances. GEMS currently has encapsulated three site-scale biogeochemical models: (1) CENTURY^58^, (2) Erosion and Deposition Carbon Model (EDCM)^32^, and (3) Land Greenhouse-Gas Accounting Tool (LGAT)^2^. GEMS can drive these models to simulate ecosystem carbon dynamics (e.g., CO_2_ and CH_4_ fluxes and changes of carbon pools) in vegetation and soil at temporal and spatial scales.

EDCM is a process-based biogeochemical model that simulates carbon and nitrogen cycles in diverse ecosystems at a monthly time step and takes into account the impacts of land management and disturbances. EDCM^32^ is a modified version of CENTURY (version IV) and uses up to 10 soil layers to simulate the SOC dynamics in the entire soil profile. An updated version of EDCM—EDCM-Auto^49^,^59^—has incorporated a generic auto-calibration package with two options: the Shuffled Complex Evolution (SCE) algorithm and the Flexible Modeling Environment (FME) R package.

The GEMS framework, as an interface and platform, is responsible for managing input and output databases and executing the encapsulated ecosystem models. The diagram of GEMS-EDCM and its execution environment for large-scale modeling can be found in Wu *et al*.^59^. Directed by GEMS, EDCM-Auto has been used to assess carbon stocks and fluxes under changing climate and land use for the baseline and projection periods across the conterminous United States^2^,^13^,^42^.

### Model inputs, setup, and implementation

GEMS-EDCM uses a variety of input data layers including climate, soil, land use, land management, and disturbances. In terms of the data coverage, all the input data layers have been setup for the conterminous United States (CONUS) in a standard format (NetCDF4), and the time-series data (e.g., climate, land use, management, and disturbance) cover a 59-year time frame from 1992 to 2050^60^. We divided this time frame into baseline (1992–2010) and projection (2011–2050) periods for the national assessment^2^,^13^ and the current study.

Soil data were mainly from Soil Survey Geographic Database (SSURGO)^61^, but the State Soil Geographic Database (STATSGO)^62^ was used when the SSURGO information was not available. The initial carbon pools—soil carbon and forest biomass—were from the national data layers of soil carbon (based on soil data from the SSURGO) and forest biomass (Forest Inventory and Analysis (FIA) unit and age-based biomass survey data) the Land Carbon Team built for 1992^42^, which was the starting year for the national assessment^13^,^42^ and the simulations in this study.

Climate and land-use data are two key drivers to project carbon dynamics. For climate, we used monthly precipitation and air temperature data from the Parameter-elevation Regressions on Independent Slopes Model (PRISM) for the baseline period. GCM projection data by the Model for Interdisciplinary Research on Comate (MIROC 3.2)^63^ under three Intergovernmental Panel on Climate Change (IPCC) scenarios (A1B, A2, and B1) were used for the projection period. A previous study, which compared data between 1970–2010 and 2011–2050, demonstrated an increase in air temperature (about 2.4 °C) and little change in precipitation for scenario B1; whereas the climate seemed to be a little drier under scenario A2^42^. For the required annual land-use data, we used the simulated 250-m products of the FORecasting SCEnarios of future (FORE-SCE) land-use model^64^ under the same three IPCC scenarios, as shown in Figure S.1 in the Supporting Information. The FORE-SCE croplands were further spatially allocated for specific crop species (e.g. corn, soybean) as described by Schmidt *et al*.^60^. Details of land-use projection using FORE-SCE can be found in previous publications^33^,^34^,^65^.

For proper projection of plant growth and grain production, the EDCM model also took into account the enriched CO_2_ effects^66^ and the biological enhancement of grain production owing to gene technology, which can be found in our previous national-scale assessment^2^,^38^. Specifically, the corn yield has experienced continuous increase in the past century and will also increase but with a much lower rate for the coming decades^2^,^38^. The auto-fertilization function of the model based on N stress was implemented to ensure nitrogen content would not be a limiting factor of crop growth in this study. In addition, the corn harvest index is about 1:1^67^, indicating corn stover biomass is n equivalent to the grain yield. Because corn stover production and harvesting are the foci of the study, we presented stover biomass only in graphs and tables but it may also represent corn grain yield.

Although the spatial resolution of model implementation is 250 m, GEMS provides two options: this resolution or systematic sampling simulations. 10 × 10 sampling, for example, refers to sampling at 10-pixel interval in both horizontal and vertical directions (equivalent to 1% sampling rate). This sampling approach with 1% sampling rate can help save substantial processing time for model calibration and application without sacrificing accuracy^2^, and was also used in this study.

### Model calibration and validation

Process-based models like EDCM also contain parameters which need to be calibrated by model inversion for reasons such as the lack of field measurements, mismatch between measurement and modeling scales, and heterogeneity of the physical environment for regional modeling^68^,^69^. Actually, according to CENTURY documentation^66^, the plant production is a function of genetic maximum potential production for each plant (named as PRDX), temperature, soil moisture, nutrients, and other species-specific parameters (e.g., Maximum leaf area index, optimal temperature, carbon allocation, as included in file crop.100). Among these factors, PRDX has both genetic and environmental components, and thus it is the foremost parameter for controlling the production for a given species and a given environment and it will be frequently used to calibrate the crop production^66^. For EDCM (the modified version of CENTURY), therefore, we selected this most sensitive parameter (PRDX) to calibrate the plant production for different species, environments, and varieties^66^. For this calibration, we used the observed grain yield for croplands (e.g., corn and soybean) and the Moderate-Resolution Imaging Spectroradiometer (MODIS) net primary production (NPP) for non-croplands (e.g., forest and grasslands). Because of its more efficient implementation, the SCE algorithm of EDCM-Auto^49^,^59^ was selected to perform the parameter optimization procedure. As in the national-scale assessment from the USGS Land Carbon Project^2^,^13^, the same 9-year (2001–2009) county-based grain yield data and 10-year (2001–2010) pixel-based NPP data were used for model calibration and validation^59^, with the first five years for calibration and the subsequent years for validation. To evaluate the model performance for the calibration and validation periods, we used three common criteria, including Percent Bias (PB), Coefficient of Determination (R^2^), and Root Mean Square Error (RMSE).

### Scenario setting

To predict potential impacts of climate, land-use change, and corn stover harvesting on SOC dynamics for the projection period (2011–2050), we used projected atmospheric CO_2_ concentrations and climate forcing by MIROC, land-use by FORE-SCE, and crop species information under the three IPCC scenarios (A1B, A2, B1) as inputs to drive the EDCM model. Corn stover were considered as cellulosic feedstock for biofuel production; however, it is not possible to remove 100% of the stover produced partly because of the mechanical techniques and environmental protection (e.g., soil and water conservation, soil fertility)^70^,^71^. Therefore, we proposed three potential corn stover harvesting schemes—30%, 50%, and 70% of corn stover removal rates. As a result, there are nine modeling scenarios covering all the combinations of climate, land use, and stover harvesting rates (see Table 2).

## Additional Information

**How to cite this article**: Wu, Y. *et al*. Projection of corn production and stover-harvesting impacts on soil organic carbon dynamics in the U.S. Temperate Prairies. *Sci. Rep*. **5**, 10830; doi: 10.1038/srep10830 (2015).

## Supplementary Material

Supplementary Information

## Figures and Tables

**Table 1:** Corn stover biomass and SOC content in 2010 and their projections in 2050.

Time	IPCC scenario and corn stover removal rate		Corn planting area (10^5^ km^2^)	Annual production of corn stover biomass		Corn field SOC		Regional SOC	
				Capacity (TgC/yr)	Density (kg C/m^2^/yr)	Storage (TgC)	Density (kg C/m^2^)	Storage (TgC)	Density (kg C/m^2^)
2010	-		1.15	52.2	0.45	724.0	6.31	3113.1	5.97
2050	A1B	30%	1.48	104.7	0.71	1044.4	7.04	3508.9	6.73
		50%				1009.1	6.81	3448.8	6.61
		70%				975.3	6.58	3390.9	6.50
	A2	30%	1.39	88.7	0.64	983.6	7.08	3521.4	6.75
		50%				951.9	6.86	3464.7	6.64
		70%				921.5	6.64	3410.0	6.54
	B1	30%	1.40	96.3	0.69	983.6	7.04	3486.0	6.69
		50%				951.7	6.81	3430.5	6.58
		70%				920.7	6.59	3376.6	6.48

**Table 2:** Definition of scenario terms for future period (2011–2050) in this study.

No.	Scenario term	Description*
1	A1B_30	A1B climate and land cover with a corn stover removal rate of 30%
2	A1B_50	A1B climate and land cover with a corn stover removal rate of 50%
3	A1B_70	A1B climate and land cover with a corn stover removal rate of 70%
4	A2_30	A2 climate and land cover with a corn stover removal rate of 30%
5	A2_50	A2 climate and land cover with a corn stover removal rate of 50%
6	A2_70	A2 climate and land cover with a corn stover removal rate of 70%
7	B1_30	B1 climate and land cover with a corn stover removal rate of 30%
8	B1_50	B1 climate and land cover with a corn stover removal rate of 50%
9	B1_70	B1 climate and land cover with a corn stover removal rate of 70%

Note:

^*^Climate data (precipitation and air temperature) were from projections by MIROC.
