# Supplementary material for: Projection of corn production and stover-harvesting impacts on soil organic carbon dynamics in the U.S. Temperate Prairies
Source: Sci Rep. 2015 Jun 1;5:10830. doi: 10.1038/srep10830 (PMC4450590; doi:10.1038/srep10830)
Supplement: Supplementary Information [file srep10830-s1.pdf]

**Supporting Information**

**Manuscript Title:**

Projection of corn production and stover-harvesting impacts on soil organic dynamics in the U.S. Temperate Prairies

**Authors:**

Yiping Wu[1*], Shuguang Liu[2*], Claudia J. Young[3], Devendra Dahal[4], Terry L. Sohl[2], Brian Davis[4]

[1] ASRC Federal, contractor to U.S. Geological Survey (USGS) Earth Resources Observation and Science (EROS) Center, Sioux Falls, SD 57198, USA

Email: ywu@usgs.gov (Y. Wu)

[2] U.S. Geological Survey (USGS) Earth Resources Observation and Science (EROS) Center, Sioux Falls, SD 57198, USA

Email: sliu@usgs.gov (S. Liu)

[3] Innovate! Inc., contractor to the USGS EROS Center, Sioux Falls, SD 57198

[4] Stinger Ghaffarian Technologies, contractor to the USGS EROS Center, Sioux Falls, SD 57198

**Projected land cover trend**

Based on the FORE-SCE land cover model projections, the annual proportions of land-cover classes during 2001 through 2050 under the four IPCC scenarios (A1B, A2, B1, and B2) are presented below.

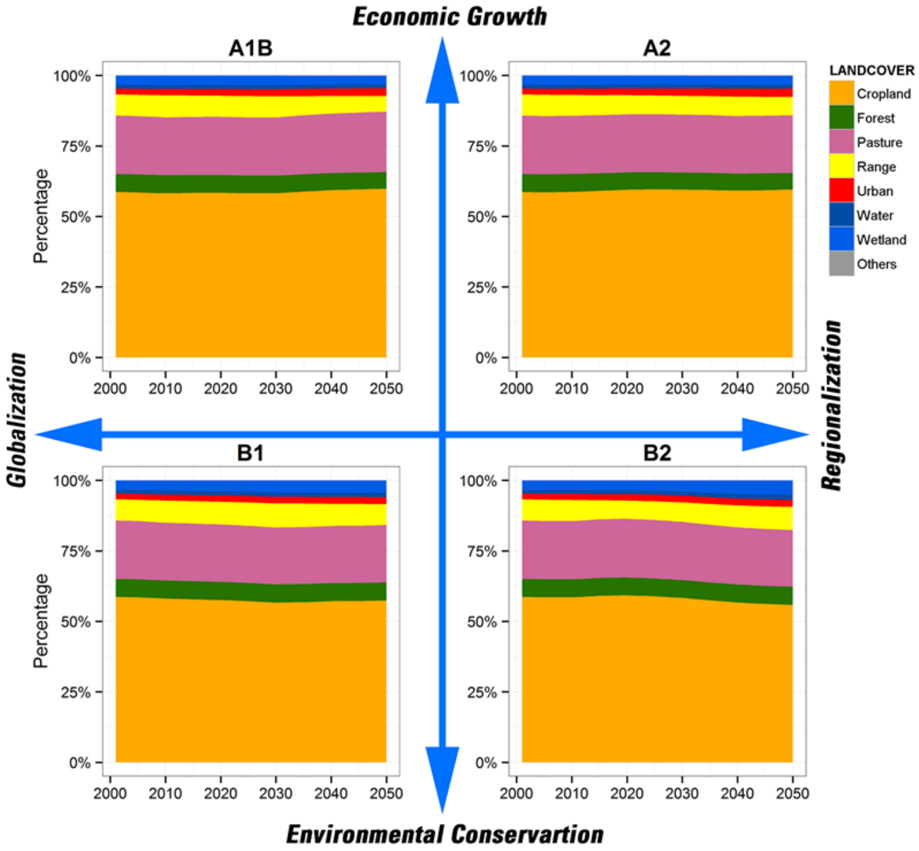


Figure S.1 Annual time series of land-cover class proportions in the Temperate Prairies region from 2001 to 2050 under the four scenarios (A1B, A2, B1, and B2).

**Location of the study area**

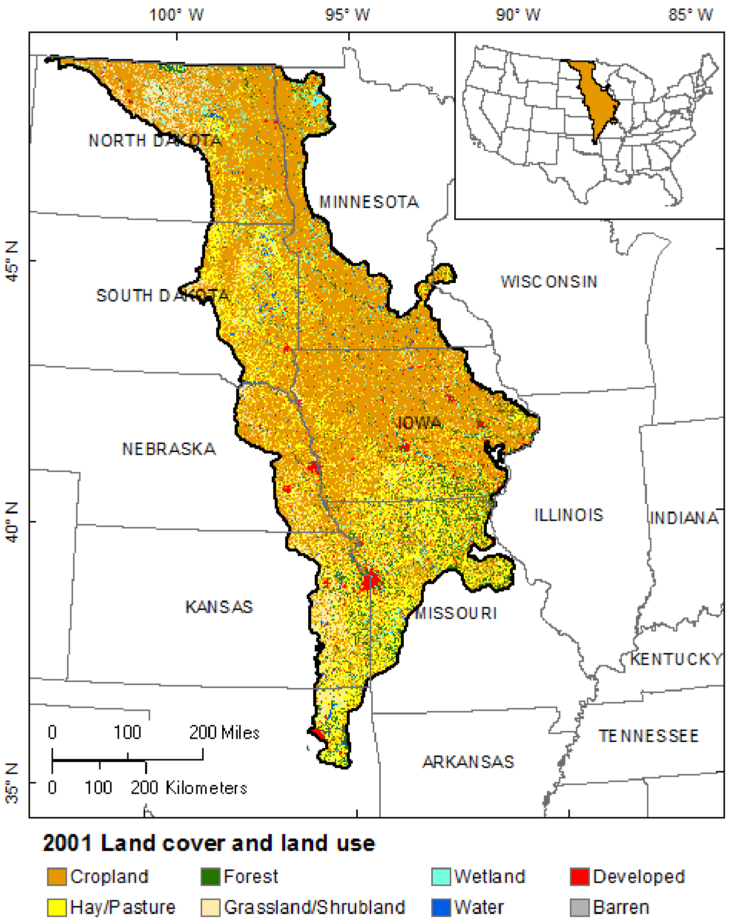


Figure S.2 Location and the land cover and land use map (2001) of the Temperate Prairies (Ecoregion 9.2). The map was created using ArcGIS10.2.
